# Experimental implantoplasty outcomes correlate with fibroblast growth in vitro

**DOI:** 10.1186/s12903-020-1012-1

**Published:** 2020-01-30

**Authors:** Mehrnaz Beheshti Maal, Stig Aanerød Ellingsen, Janne Elin Reseland, Anders Verket

**Affiliations:** 1 Department of Biomaterials, Institute of Clinical Dentistry, University of Oslo, Oslo, Norway; 2 Department of Periodontology, Institute of Clinical Dentistry, University of Oslo, Geitemyrsveien 69-71, P.O.Box 1109 Blindern, NO-0317 Oslo, Norway

**Keywords:** Implantoplasty, Dental implant, In vitro, Fibroblast, Implant surface

## Abstract

**Background:**

Implantoplasty is an option in peri-implantitis treatment, but little is known about the effect on the soft tissue. The aim of the study was to characterize surface roughness following experimental implantoplasty and to examine its effect on human fibroblast growth and secretion of selected proteins.

**Methods:**

Titanium grade IV coins were mechanically treated with six different rotating bur sequences; diamond burs or carbide burs alone, or followed by either Arkansas stone bur or silicone burs. Machined and rough-surface sandblasted, acid-etched (SLA) coins were used as control. The surface topography was characterized by scanning electron microscope and profilometer. Human gingival fibroblasts from two donors were cultured on the coins to quantify the effect on cell morphology, growth, and protein secretion by confocal microscopy and multiplex immunoassay.

**Results:**

All surface roughness parameters were lower for the surfaces treated with experimental implantoplasty than for the SLA surface, and the sequence of carbide burs followed by silicone burs rendered the least rough surface of the test groups. The implantoplasty procedures changed the elemental composition of the titanium surface. High surface roughness showed a weak to moderate negative correlation to fibroblast growth, but induced a higher secretion of VEGF, IL-6 and MCP-3 to the cell medium compared to the least rough surfaces of the test groups. At day 30 fibronectin levels were higher in the SLA group.

**Conclusions:**

The surface roughness following implantoplasty demonstrated a weak to moderate negative correlation with the growth of fibroblasts. The addition of Arkansas stone and silicon burs to the experimental implantoplasty bur protocol rendered an initial increase in fibroblast growth. Implantoplasty altered the elemental composition of the titanium surface, and had an effect on the fibroblast cytokine secretion and fibronectin levels.

## Background

Peri-implantitis is a biofilm-mediated progressive inflammatory disease in the tissues surrounding the dental implant, which ultimately may lead to its loss [1]. To date there is no consensus on a treatment protocol for peri-implant diseases. Therapies researched have mostly been modifications of periodontitis treatment modalities [2].

In 1990, Lozada and co-workers presented a case report where peri-implantitis was treated by open flap debridement in addition to recontouring the exposed implant surface with high-speed diamond and aluminum oxide burs [3], a procedure now referred to as implantoplasty. The advantage of a smooth versus a rough surface is facilitated oral hygiene and a reduction in bacterial colony forming units [4].

A previous clinical trial compared open flap debridement with or without implantoplasty. No change in radiographic bone loss from baseline to the 3-year follow-up was observed in the test group, whereas a mean marginal bone loss of 1,44 mm was found in the control group [5]. Another clinical trial has been performed as well, albeit not comparing groups with and without implantoplasty. Schwarz and co-workers did implantoplasty on 38 patients as part of a treatment with and without the use of Er-YAG laser [6]. These limited studies have presented promising clinical outcomes following implantoplasty.

Rimondini et al. investigated in vitro differences in the topographical surface roughness parameters between different implantoplasty bur protocols. All bur sequences tested rendered surfaces that were smoother than the plasma-spray-coated control implant, whereas no significant differences were found between the different bur sequences and the machined control [7]. More recent in vitro studies have focused on the optimum bur sequence [8–11], the heat generation following titanium polishing [11–13], and biocompatibility [14, 15]. Fracture resistance has also been the focus of in vitro studies [16], but according to a recent systematic review no fractures following implantoplasty have been reported in the literature [17].

A possible advantage of implantoplasty in addition to facilitated oral hygiene is a potential improvement of the soft tissue adaptation to the dental implant. An ideal implant surface should impede bacteria and biofilm growth and adhesion while at the same time allow rapid connective tissue attachment. A significant impact of the surface topography on connective tissue attachment has been demonstrated [18]. Previous studies have shown that human gingival fibroblasts (HGF) spread more readily on smooth as compared to rough surfaces, and that the connective tissue adhesion is affected by surface properties [19–23]. More knowledge of the mechanisms involved in the re-establishment of a soft tissue seal, of which fibroblasts play a key role, subsequent to implantoplasty treatment, is warranted. Therefore it is of interest to explore how the surface modifications made by clinicians during implantoplasty may affect HGFs.

The aim of the present in vitro study was to characterize the substrate topographies following experimental implantoplasty and to examine the fibroblast growth, attachment, morphology and cytokine secretion following culture on the various titanium substrates. The null hypothesis was that surface modifications by experimental implantoplasty have no effect on fibroblast growth, attachment, morphology or cytokine secretion.

## Methods

### Titanium coin preparation

Grade IV titanium coins, Ø 6 mm and height 2 mm were washed in five steps as previously described [24], before the surface modification procedure. The titanium coins were divided into 6 different test groups according to the sequence of burs used for experimental implantoplasty (Table 1); carbide cutting burs (CB), CB + Arkansas stone (CB + Ark), CB + Brownie and Greenie silicone burs (CB + BG), diamond burs (DB), DB + Ark and DB + BG. Two control groups were also included; coins with a sandblasted and acid-etched surface (SLA) (kindly provided by Straumann, Straumann Holding AG, Basel, Switzerland) and polished coins (P) according to a procedure previously published [24]. All burs were in contact with the titanium coin for 1 min under copious water irrigation. By-products in the irrigation water were collected using a filter paper and vacuum suction (595 Filter Paper Circles, GE Healthcare, Merck KGaA, Darmstadt, Germany). Following in vitro experimental implantoplasty procedures, all coins were rinsed with deionized water, agitated for 60 min and then autoclaved at 121 °C.

**Table 1:** Explanation of burs used for implantoplasty in each sequence

Abbreviation	Description	Manufacturer	Handpiece/Rounds per minute	Total time bur on coin per group
CB	Carbide cutting burs, red (normal toothing) and white (fine toothing)	Komet Dental, Gebr. Brasseler GmbH & Co. KG, Lemgo, Germany	Air-driven turbine handpiece 200,000 rpm + Contra angle handpiece 15,000 rpm	2 min
CB + Ark	Carbide cutting burs, red (normal toothing) and white (fine toothing) + Arkansas stone 661	(Komet Dental)	Air-driven turbine handpiece + Contra angle handpiece 15,000 rpm	3 min
CB + BG	Carbide cutting burs red (normal) and white (fine toothing) + Silicone cup brownie 030 + Silicone cup greenie 030	(Komet Dental) + Shofu, Shofu Dental GmbH, Ratingen, Germany	Air-driven turbine handpiece + Contra angle handpiece 15,000 rpm	4 min
DB	Diamond sequence of decreasing coarseness 105 μm, 40 μm, 8 μm	105 μm (250.014, Horico Dental, Hopf, Ringleb & Co. GmbH & CIE, Berlin, Germany), 40 μm (8379EF.314, Komet Dental), 8 μm (863UF.314, Komet Dental)	Air-driven turbine handpiece + Contra angle handpiece 15,000 rpm	3 min
DB + Ark	Diamond sequence of decreasing coarseness 105 μm, 40 μm, 8 μm + Arkansas stone 661	105 μm (Horico Dental), 40 μm (Komet Dental), 8 μm (Komet Dental) + (Komet Dental)	Air-driven turbine handpiece + Contra angle handpiece 15,000 rpm	4 min
DB + BG	Diamond sequence of decreasing coarseness 105 μm, 40 μm, 8 μm + Silicone cups brownie 030 + Silicone cup greenie 030	105 μm (Horico Dental), 40 μm (Komet Dental), 8 μm (Komet Dental) + (Shofu)	Air-driven turbine handpiece + Contra angle handpiece 15,000 rpm	5 min

### Surface characterizations

#### Profilometer

A total of 48 coins (*n* = 6 from each group) were analysed with a profilometer (Sensofar SensoSCAN 6.2, Terrassa, Spain). Topographical parameters were obtained using a blue light laser profilometer with a 150 × 0,95 DI Nikon objective. An arbitrary area of 292 μm × 220 μm was scanned for each coin. The surface amplitude parameters; arithmetical-mean-height (S_a_), ten-point-height of the surface (S_z_), root-mean-square deviation (S_q_), and the reduced peak height (S_pk_) values were calculated using the SensoMap software (SensoMap Standard 7.3.7690, Sensofar, Terrassa, Spain).

#### SEM and EDX

A total of 48 coins (*n* = 6 from each group), and debris from each bur-sequence procedure were analysed with a scanning electron microscope TM3030 (Hitachi High-Technologies Europe GmbH, Krefeld, Germany). The samples were mounted on an aluminum holder with carbon tape and copper conductive tape. Scanning electron microscope (SEM) images were obtained with backscattered electrons at 15 kV voltage. Furthermore, energy dispersive x-ray spectroscopy (EDX)(Quantax 70, Bruker, Billerica, USA) was used for detection of chemical elements measured in atomic percentage on the titanium coin surfaces [25].

#### Experimental in vitro design

Commercially available HGFs from two different donors (Provitro, German type Culture Collection, Berlin, Germany, Passage 6) were cultured in fibroblast growth medium (Basal medium, Provitro) supplemented with 10% fetal bovine serum, 100 U/mL penicillin, and 100 mg/mL streptomycin (GE Healthcare, Utah, USA) at 37 C° in a humidified atmosphere with 5% CO_2_. The coins (*n* = 6–10 for donor 1, *n* = 5 for donor 2 for each experimental groups) were placed into 96-well tissue culture plates (Tissue culture plates, 96 wells, VWR®, Radnor, USA). With the use of an electronic counter (Countess, Invitrogen, Carlsbad, CA, USA), cells from both donors were seeded onto the coins with a cell-number of 2000 cells/ml (~ 70 cells/coin) on the coins to be harvested at day 3 and 6, and a cell-number of 10,000 cells/ml (~ 350 cells/coin) for coins harvested after 15 days and 30 days of incubation. The same number of cells was cultured on plastic in order to monitor the cell secretion.

Cell culture media was harvested from the wells cultured with the highest cell seeding density (350 cells/coin)(*n* = 6–10 for donor 1, *n* = 5 for donor 2) every third day for the whole study period and stored at − 20 °C prior to analysis of selected cytokines secreted (Luminex assay).

#### Luminex analysis

Multianalyte profiling of the level of the markers fibroblast growth factor 2 (FGF-2), epidermal growth factor (EGF), interleukin 6 (IL-6), interleukin 7 (IL-7), interleukin 10 (IL-10), vascular endothelial growth factor (VEGF), monocyte chemotactic protein-1 (MCP-1), monocyte chemotactic protein-3 (MCP-3), interferon gamma-induced protein 10 (IP-10)(Human Cytokine/Chemokine Magnetic Bead Panel kit)(Billerica, MA, USA) in the harvested cell culture media was performed on the Luminex-200 (Luminex, Austin, TX, USA) using the Human Cytokine/Chemokine Magnetic Bead Panel kit (Billerica, MA, USA) according to the manufacture’s protocol.

#### Immunostaining

Cells cultured on coins for 3, 6, 15 and 30 days, respectively, were fixed with 4% paraformaldehyde for 20 min at room temperature. The cells were permeabilized with 0,02% Triton X-100 in PBS for 10 min at room temperature. Blocking of unspecific binding of antibodies was performed with a solution of 10% goat serum in PBS for at least one hour at room temperature. The cells were incubated overnight at 4 C with primary antibodies. Antibodies against Vinculin (1:600, #V9131, Sigma Aldrich) and Fibronectin (1:600, #F3648, Sigma Aldrich) both diluted in PBS with 2% goat serum were used. As secondary antibodies, goat-anti-mouse-Alexa647 (1:100, #A21236, Invitrogen) and goat-anti-rabbit-Alexa568 (1:100, #A11011, Invitrogen) diluted in PBS with 4% goat serum were used. To visualize the actin filaments, the cells were stained with 2,5% Phalloidin-Alexa 488 (#A12379, Invitrogen) in PBS for 20 min. The cell nucleus was stained using a solution of DAPI or Hoechst (0.3 μM)(#33,342, Thermo Scientific™) in PBS for 30 min was used. The cells were stored at 4 °C for later imaging with confocal microscopy.

#### Confocal microscopy

Cells were imaged at minimum three non-overlapping areas (554,65 × 554,65 μm) using a 20x/0,40 HCX APO CS water-immersion objective (Leica SP8, Wetzlar, Germany). Samples were exited with lasers at 405 nm, 488 nm and 552 nm. Confocal Z-stacks were used in every case. Image analysis, fibronectin quantification and cell counting was performed using ImageJ (Fiji software, 64 bit, Windows) [26]. To quantify fibronectin a dichotomous red color contrast to black threshold was arbitrarily set for each image by comparing to the original confocal images, after which the area percentage of the stain was quantified.

#### Statistical analysis

To enable comparison of secreted factors and cell growth for each of the donors, data were adjusted for cell number and calculated relative to the rough control (SLA) at each time point. The statistical analysis of data from each donor was performed in SigmaPlot (Systat Software, Inc., San Jose California, USA). Differences between experimental groups and control groups were determined using One-Way ANOVA on ranks. To facilitate comparison to other studies all figures are however presented with mean values ± standard deviation (SD). Correlation analyses were performed using Spearman correlation. A *P*-value < 0.05 was considered statistically significant.

## Results

### Experimental implantoplasty characterization

**Fig. 1:**
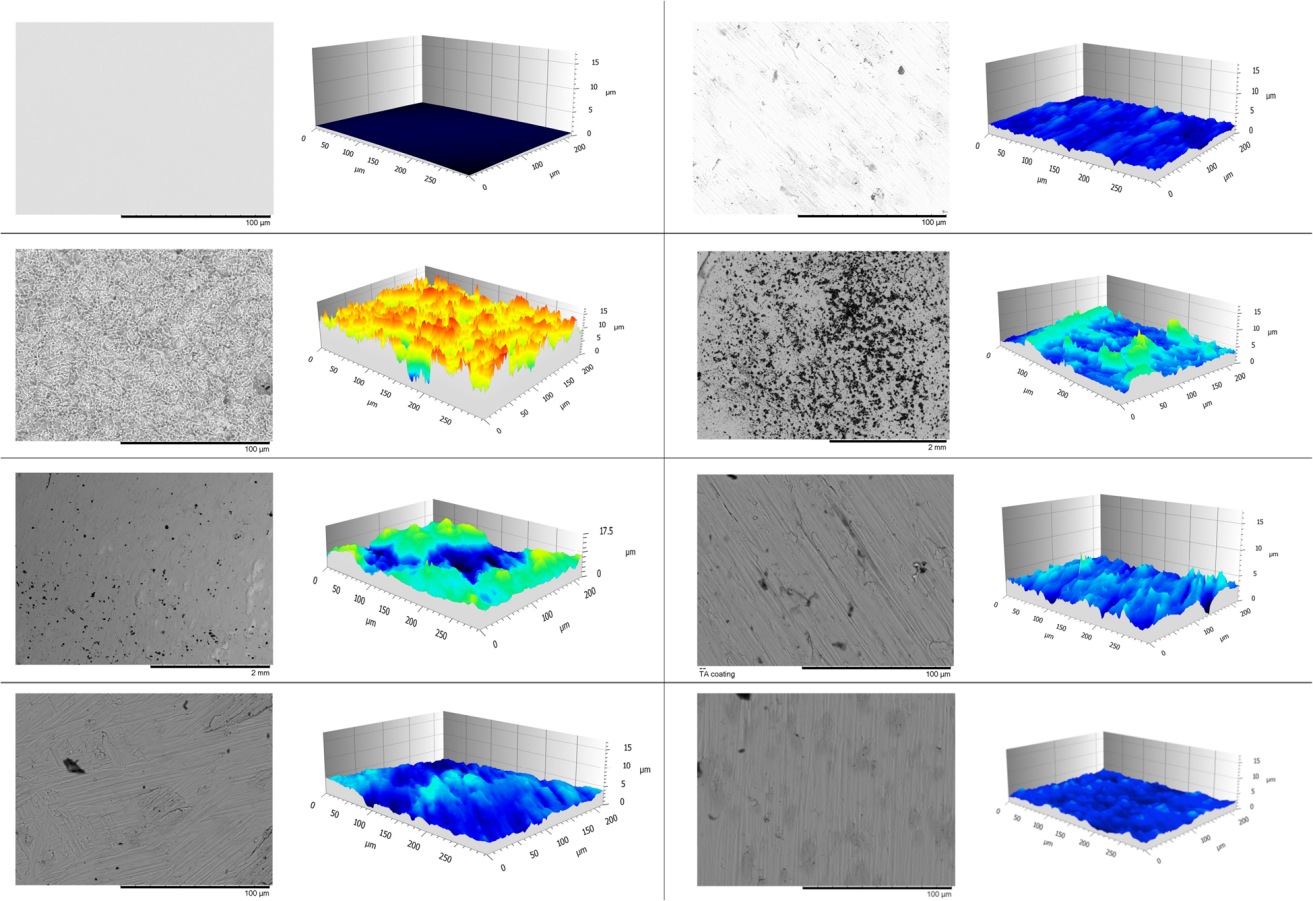
Each group represented with a SEM picture (left) and a three-dimensional profilometer profile (right). Left column from top to bottom; Polished, SLA, DB and DB+Ark. Right column from top to bottom; DB+BG, CB, CB+ARK and CB + BG

**Fig. 2:**
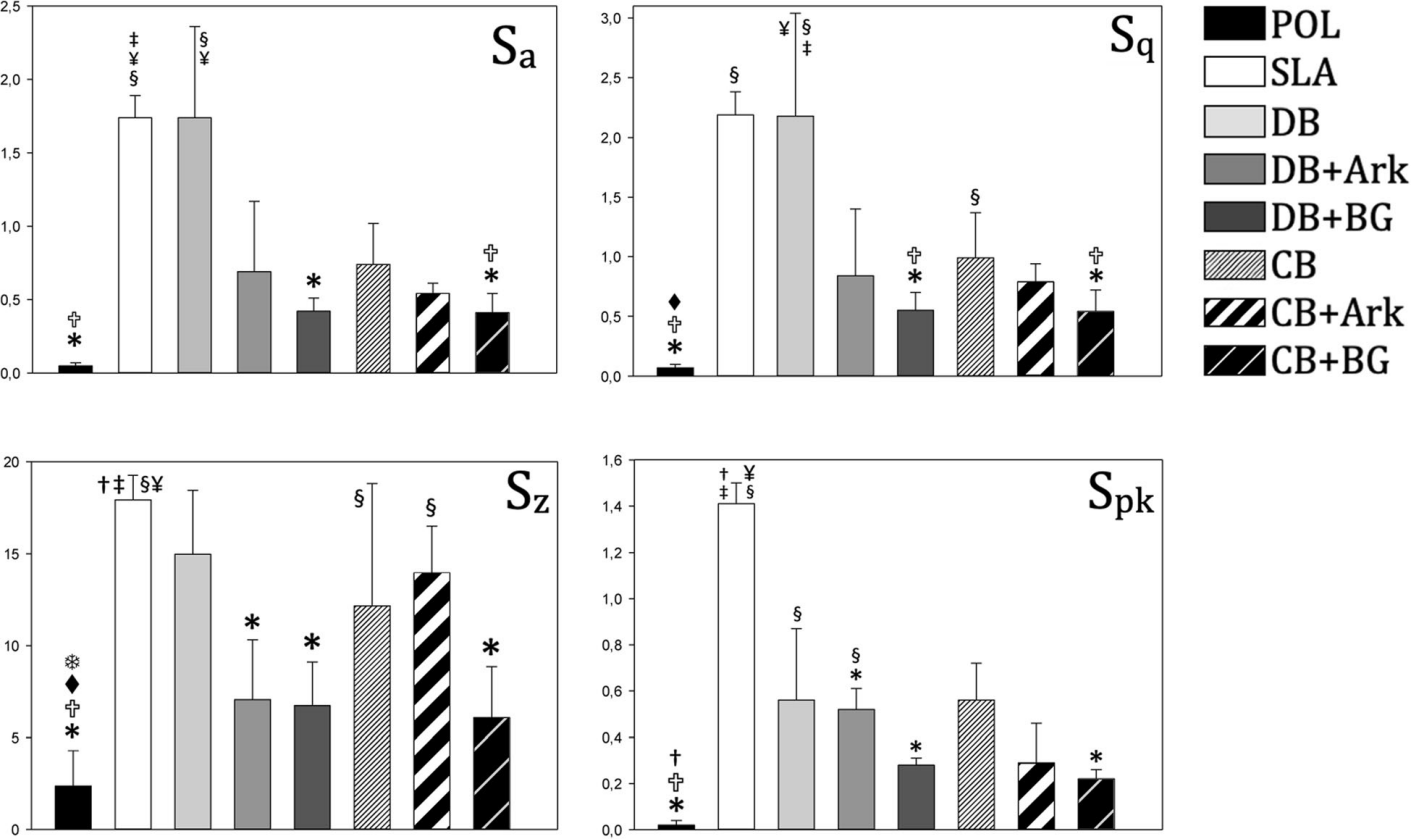
The surface topography parameters for each group showing mean values and standard deviation. Arithmetical-mean-height = (S_a_), Ten-point-height of the surface = (S_z_), Root-mean-square deviation = (S_q_), Reduced peak height (S_pk_). §statistically significantly different to P, *statistically significantly different to SLA, statistically significantly different to DB, †statistically significantly different to DB + Ark, ‡statistically significantly different to DB + BG, statistically significantly different to CB, ❄ statistically significantly different to CB + Ark, ¥statistically significantly different to CB + BG (*n* = 6)

The mean S_a_ and the S_q_ values were lower with the addition of Ark than with DB and CB alone, whereas the addition of BG rendered the lowest values among test groups (Figs. 1 and 2). The CB sequences alone or in combination demonstrated lower S_a_ and S_q_ values than the corresponding DB sequences. The coins in the DB group had a significantly higher S_a_ value as compared to POL (*P* < 0.001) and CB + BG (*P* = 0.028). SLA had a significantly higher S_a_ value as compared to POL (*P* < 0.001), CB + BG (*P* = 0.014) and DB + BG (*P* = 0.028).

The S_z_ value was significantly higher in the SLA group as compared to the POL (*P* < 0.001), CB + BG (*P* = 0.012), DB + Ark (*P* = 0.050) and DB + BG groups, respectively (*P* = 0.023). DB induced a higher S_z_ value than POL (*P* = 0.005), whereas both CB and CB + Ark were significantly higher than POL (*P* = 0.016; *P* = 0,002), respectively (Fig. 2). The S_pk_ values for SLA were significantly higher than POL (*P* < 0.001), DB + BG (*P* = 0.002), DB + Ark (*P* = 0.007) and CB + BG (*P* = 0.030). DB and DB + Ark were significantly higher than POL (*P* = 0.005; *P* = 0.014), respectively (Fig. 2).

The different bur protocols led to different surface structures as demonstrated by the SEM micrographs (Fig. 1). The CB-including sequences were covered by debris visible as dark spots covering the surface, and the amount of debris was less when CB was combined with either Ark or BG. Minor debris could also be observed on the DB-treated coins. Overall, the DB-including sequences had more irregular and non-linear grooves when compared to CB. Addition of Ark or BG made the irregularities and the grooves less pronounced for both DB and CB sequences.

**Fig. 3:**
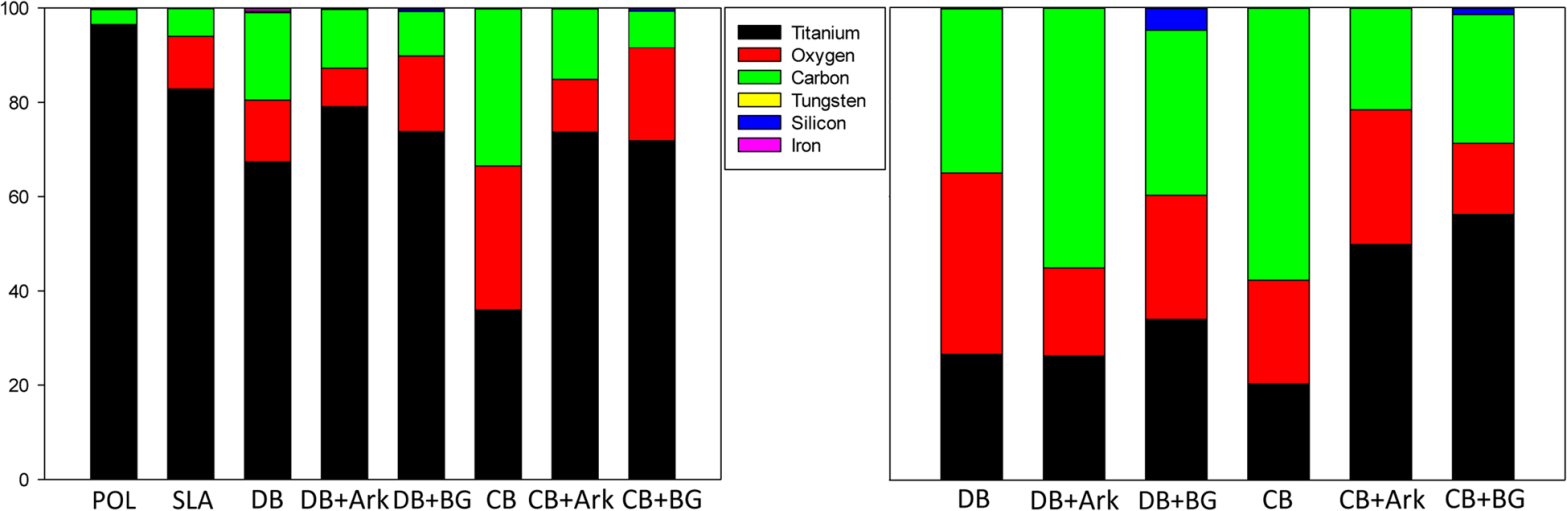
The proportions of the elements titanium, oxygen, carbon, tungsten, silicon and iron (at.%) on the coin surfaces following implantoplasty and in the debris by-products. The vertical axis represents the atomic percent of the respective elements from 0 to 100% (*n* = 6)

The EDX analysis (Fig. 3) demonstrated that the percentage of carbon (C) decreased with the addition of Ark and BG to both the DB- and CB-sequences. More oxygen (O) was detected on the surface following the use of BG as compared to Ark. Significantly more titanium (Ti) was detected in the SLA (*P* = 0.004), untreated (*P* = 0.002) and POL (*P* < 0.001) groups compared to the CB group. POL also showed significantly more Ti than DB (*P* = 0.025). O was found in significantly higher amounts in CB (*P* = 0.005), (*P* = 0.012) and CB + BG (*P* = 0.003), (*P* = 0.008) as compared to POL and untreated coins, respectively. C was found in significantly higher amounts in CB when compared to SLA (*P* = 0.004), untreated (*P* = < 0.001) and POL (*P* < 0.001). DB also had significantly more C than both untreated (*P* = 0.020) and POL (*P* = 0.006). DB + Ark had more C than POL (*P* = 0.029). The BG sequences had the highest values of silicone (Si) with the DB + BG sequence with significantly more Si than SLA (*P* < 0.001), CB (*P* = 0.002), DB (*P* = 0.046) and CB + Ark (*P* = 0.049). CB + BG had significantly more (Si) than SLA (*P* < 0.001) and CB (*P* = 0.003). Iron (Fe) and tungsten (W) were detected in low percentages but not different between groups.

Only one sample of debris from each group could be analysed because a certain quantity was required for the EDX-analysis (Fig. 3). Si was detected in higher proportions in the bur-sequences that included BG. The proportion of Ti in the by-products increased with the addition of Ark, but was the highest for CB- and DB-sequences combined with BG.

### Fibroblast response to surface treatment

**Fig. 4:**
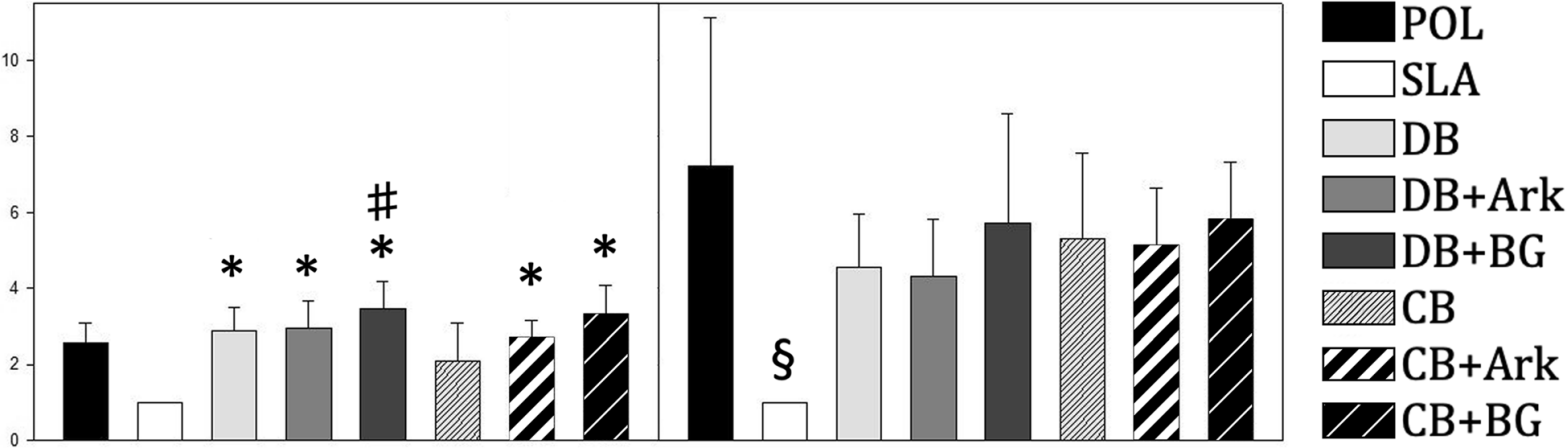
Cells per area relative to the cell number of the SLA control (SLA = 1.0) at day 3 (left) and day 6 (right). Data for days 15 and 30 are not shown. * significantly higher than SLA. # significantly higher than CB. § significantly lower than all other groups (*n* = 5 per donor)

The number of fibroblasts was increased in all groups except for in the SLA group (Fig. 4). Significantly more fibroblasts were found on the surface treated with DB + BG burs as compared to CB (*P* = 0.034) at day 3, and SLA had significantly less fibroblasts as compared to all other groups except for POL and CB (*P* < 0.04). On day 6, SLA had less fibroblasts than all other groups except DB + Ark (*P* < 0.032). Beyond day 6 the differences between SLA and the other groups continued to increase, and there were significantly more cells in all other groups than the SLA group at both day 15 (*P* < 0.001) and day 30 (*P* < 0.001).

A significant correlation between the S_a_ values and number of cells were found at day 3 (*P* = 0.001), 6 (*P* = 0.001), 15 (*P* = 0.004), and 30 (*P* = 0.002) (Table 2).

**Table 2:** Table demonstrating the correlation between cell numbers and S_a_ values

Sample	Correlation Coefficient	*P*-Value
Day 3 (*n* = 72)	−0.296	0.001
Day 6 (*n* = 74)	−0.358	0.001
Day 15 (donor 2) (*n* = 34)	−0.487	0.004
Day 30 (donor 1) (*n* = 39)	−0.473	0.002

**Fig. 5:**
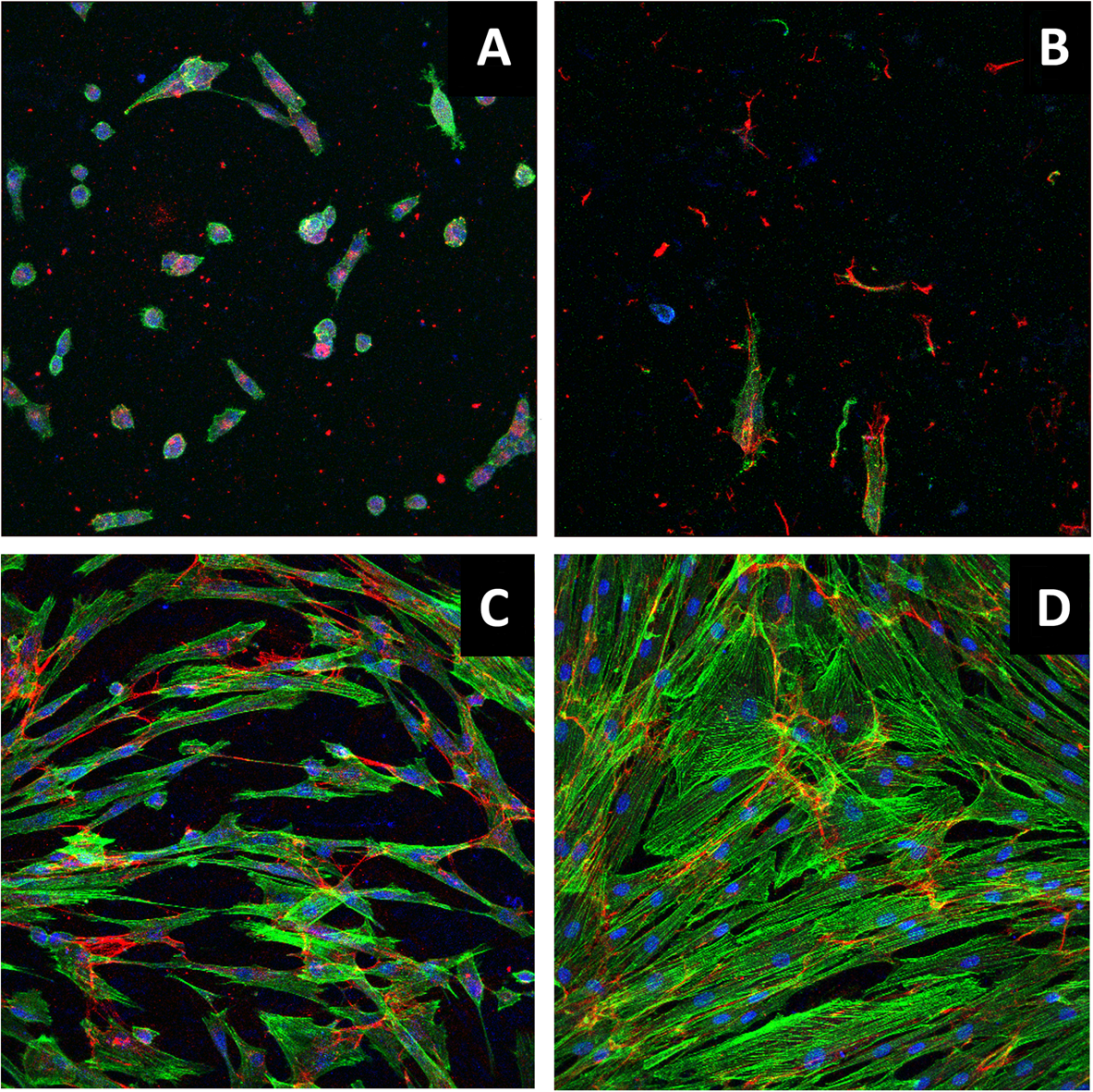
Representative images of confocal microscopy. Fibronectin (red), DAPI (blue), and phalloidin (green). Images **a** (SLA group day 3), **b** (SLA group day 30), **c** (POL day 3), and **d** (DB + BG day 6)

In general, the fibroblasts were found to be large and with a clear elongated shape on all surfaces except for cells cultured on the SLA-surface; here they were more round-shaped at day 3 (Fig. 5). The cells had ovoid nuclei in all groups, which became smaller around day 30. The cells were oriented in parallel to each other in all groups except for in the SLA group where cells were either single or in separated clusters. There was a clear phalloidin exhibition of elongated actin-filaments in every group with the exception of SLA where the cells had shorter filaments with unclear orientation.

**Fig. 6:**
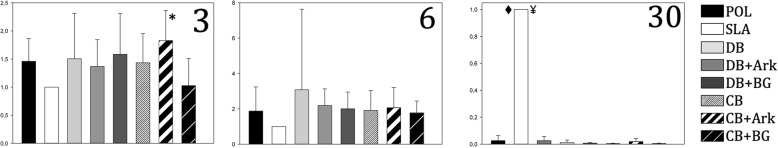
Fibronectin levels relative to the SLA control at day 3, 6 and 30. (*n* = 5 per donor) *statistically significantly different to SLA, statistically significantly different to CB, ¥statistically significantly different to CB + BG

Fibronectin levels identified by immunostaining corresponded to the amount of cells of the various surfaces in all groups (Fig. 5). A higher level of fibronectin was found on the CB + Ark coins as compared to the SLA control coins at day 3 (*P* = 0.016), whereas SLA had higher relative levels than CB (*P* = 0.042) and CB + BG (*P* = 0.03) at day 30 (Fig. 6). The signals of the antibodies against vinculin appeared to be non-specific, and could thus not be evaluated.

**Fig. 7:**
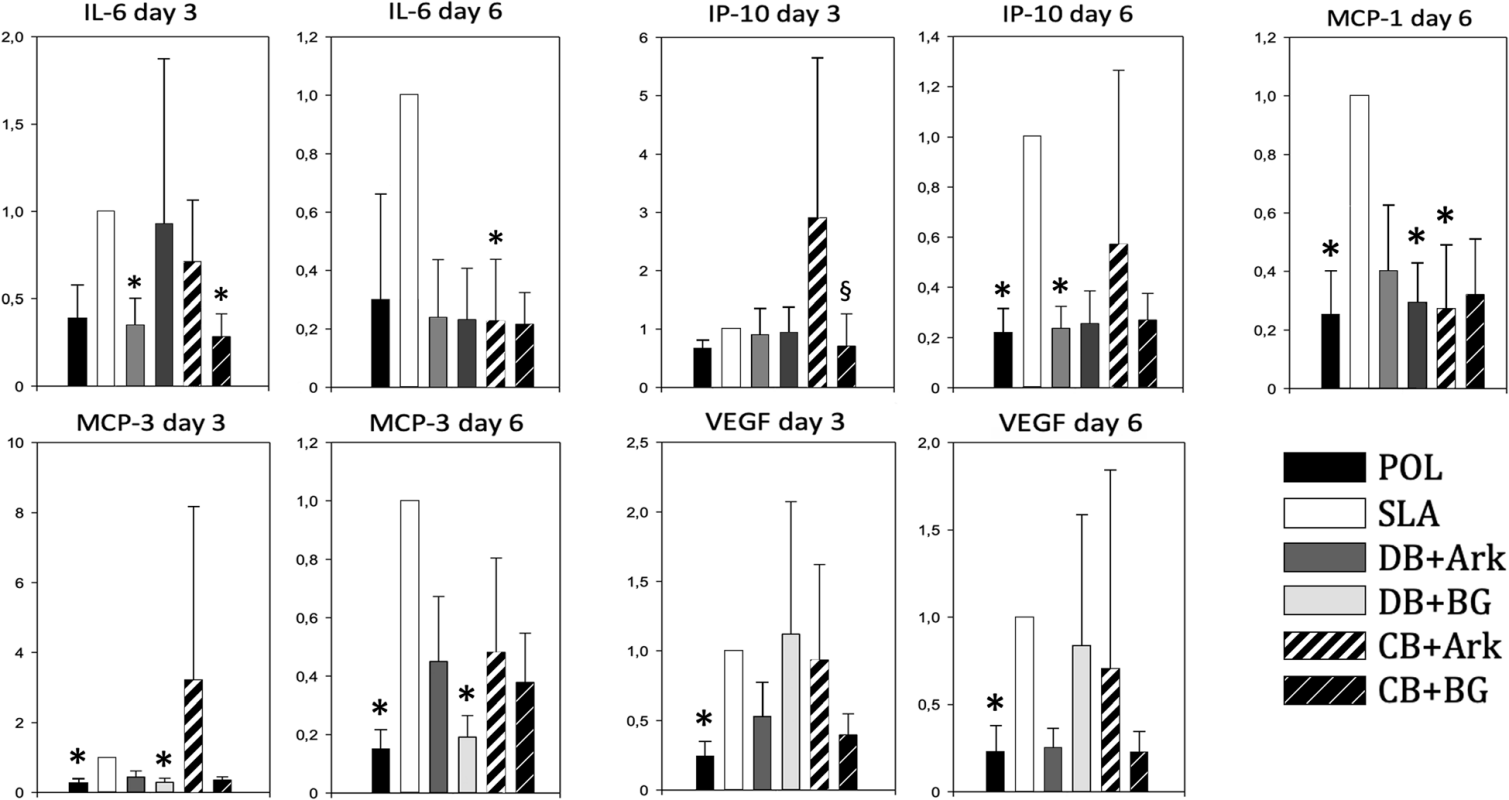
Concentrations of respective cytokines relative to the concentration of the cell medium in the SLA control (SLA = 1.0) (*n* = 3 per donor). *significantly lower than SLA. §significantly lower than CB + Ark

The concentrations of EGF, IL-7, IL-10 in the cell medium were for many samples below the detection level for the kits and were consequently not considered here. A significantly higher concentration of IL-6 in the cell medium was found from HGFs cultured on SLA as compared to DB + Ark (*P* = 0.004) and CB + BG (*P* = 0.034) on day 3, and at day 6 it was higher on SLA as compared to CB + Ark (*P* = 0.037)(Fig. 7). At day 3, there was significantly higher concentration of IP-10 in the cell medium from HGFs cultured on CB + ARK as compared to CB + BG (*P* = 0.031), and at day 6 it was higher on SLA as compared to POL (*P* = 0.019) and DB + Ark (*P* = 0.041). There was a significantly higher concentration of MCP1 at day 6 in the cell medium from HGFs cultured on SLA as compared to CB + Ark (*P* = 0.006), POL (*P* = 0.016), DB + BG (*P* = 0.034). There was a significantly higher concentration of MCP3 in the cell medium from HGFs cultured on SLA as compared to POL and DB + BG at both day 3 and 6, respectively (*P* = 0.004)(*P* = 0.012)(*P* < 0.001)(*P* = 0.002). There was a significantly higher concentration of VEGF in the cell medium from HGFs cultured on SLA as compared to POL at day 3 (*P* = 0.002) and at day 6 (*P* = 0.043)(Fig. 7).

## Discussion

The initial growth of fibroblasts demonstrate a weak to moderate negative correlation to the surface roughness (S_a_) following a selection of experimental implantoplasty strategies. All CB sequences rendered smoother surfaces than the DB sequences, and the additional use of BG resulted in smoother surfaces than Ark when combined with both CB and the DB sequences. The different implantoplasty bur sequences affected the elemental composition of the titanium surfaces, but when comparing the secretion of IL-6, VEGF, MCP1, MCP3 and IP-10 and fibronectin levels, the rough control (SLA) in general demonstrated higher levels whereas only small differences were observed between the implantoplasty test groups.

That fibroblasts respond differently according to substrate roughness is already known [17, 18, 27]. However, this has only been shown on surfaces prepared in laboratories with delicate equipment and procedures impossible to replicate intraorally in patients. In case of peri-implantitis, surface alterations of rough implants may be desirable to facilitate hygiene measures, but potentially also to improve the soft tissue adaptation. This study is the first to demonstrate that chairside treatment with the use of only a few bur sequences has the capacity to influence subsequent in vitro fibroblast growth and adhesion. This indicates that implantoplasty treatment outcomes may affect soft tissue healing, adaptation and homeostasis, and not only the ease of microbial disruption in oral hygiene.

Experimental implantoplasty procedures including BG rendered the lowest S_a_ values, which is in agreement with Ramel and co-workers. Although they analyzed cylindrical dental implants with a two-dimensional stylus profilometer, the order of surface roughness for BG, Ark and DB as measured by R_a_ is in accordance with the present study [9]. Bollen et al. suggested that bacterial colonization is not affected as long as the substrate roughness is below Ra 0.2 μm [28]. In the present study only the POL control group had a S_a_ value below this threshold, which is in agreement with previous studies [9, 10, 13]. To the best of the authors’ knowledge, only Costa-Berengeuer and co-workers have reported S_a_ values of less than 0.2 μm by the use of mere chairside bur-sequences [16]. Potential explanations for these conflicting findings may be that Costa-Berenguer and co-workers used a high-speed handpiece and changed burs for every implant.

The DB-treated and SLA coins demonstrated very different fibroblast growth. Despite clear discrepancies in both the profilometer analysis and the SEM images, the S_a,_ S_z_ and S_q_ values were similar for coins in the DB and SLA group. This questions the validity of the use of these roughness parameters alone to determine surface roughness and clinical applicability of implantoplasty. One may hypothesize whether other surface roughness parameters or combinations of parameters would be more suitable for use in this context. In the present study the parameter S_pk_ seemed to better differentiate SLA and DB. S_pk_ represents the mean height of peaks above the core surface, and a large S_pk_ value indicates a surface of high peaks providing a small initial contact area, which may be an explanation for the poor HGF growth in the SLA group.

The numerous dark spots that covered the surface of the CB-treated coins were not visible to the same extent in the other groups. One may hypothesize these dark-spots are debris following the CB sequence. The highest percentage of Si was observed on the surface of, and in the debris from, coins treated with BG. This demonstrates that BG burs leave behind more Si than the other burs, which is not surprising as BG are silicone burs. However, it also suggests not all silicon is lost as debris but some may be found on the implant surface.

Higher numbers of fibroblasts were found in groups with lower surface roughness (S_a_ value) for both diamond and carbide sequences. However, the POL control group with the lowest S_a_ value did not have significantly more fibroblasts as compared to any of the test groups at any time point. This may indicate that fibroblast growth and adhesion was not only affected by the surface S_a_ value in the present study. The enhanced growth on the smoothest surfaces observed in the present study is in agreement with the findings from Könönen et al. who compared fibroblast proliferation on three different titanium substrates. They also found that the fibroblasts cultured on the roughest surface were round and flat and had aberrant morphology after 3 days. Other previous studies have also reported higher viability and proliferation on smoother titanium surfaces [20, 29, 30].

Studies have suggested smooth or finely grooved titanium substrates may be optimal for soft tissue adaptation due to its support of integrin-receptor clustering into focal and ECM contacts [26]. One of the main functions of focal adhesion proteins is to promote cell-attachment to the extracellular matrix [31]. These proteins are also important for cell-motility, normal cell function and interaction with the environment [32, 33]. Fibronectin is a major structural glycoprotein that contributes attachment and spreading of fibroblasts [34]. The distribution of fibronectin was investigated in the present study, but no overall trend was observed according to the different implantoplasty surface treatments at day 3 or 6. At day 30 however, the fibronectin level was higher in the SLA group. However, one must keep in mind that at day 30 very few cells were present in the SLA group whereas the fibronectin remained, which at this time point greatly affected the results presented relative to the SLA group.

Vinculin is a cytoskeletal protein involved in formation of focal adhesion [35], and for this reason we aimed to quantitatively and qualitatively analyse it. Previous studies have indicated conflicting results with respect to HGFs’ expression of vinculin [20, 36, 37].

Since the effect of the experimental implantoplasty treatment on the fibroblast growth was limited beyond day 6 in the present study, the analyses of cytokine secretion to the cell medium was performed at the two earliest time points only. Also the DB and CB sequences were left out of the Luminex analysis since these rendered the roughest surfaces of the experimental implantoplasty treatments, and would therefore not be considered in a clinical setting. The different cell densities were used to facilitate the chosen analyses. The cell seeding density of ~ 70 cells/coin were used for growth analysis, whereas the density of ~ 350 cells/coin were used for growth analysis and Luminex analysis. Wells seeded with the lower cell density were used for cell growth analysis in order to avoid early confluence due to rapid cell growth and to characterize the morphology of single isolated cells. The higher cell density was used in wells included in the Luminex assay to increase the concentration of cytokines secreted to the cell medium. One may speculate to what extent the various experimental implantoplasty substrates contribute different biological responses. For example, the concentration of IP-10 was higher in the cell medium from HGFs cultured on CB + Ark as compared to CB + BG at day 3. Whether such findings have any clinical relevance must be addressed in in vivo studies and clinical research. In the Luminex assay, a limited set of factors known to be expressed and secreted by fibroblasts that have potential stimulatory and/or inhibitory effects on surrounding cells and soft tissue in vivo, and/or potential implications in bone metabolism, was chosen.

Fibroblast adhesion and growth is only one of few events taking place following implantoplasty-treatment. The epithelial and in vivo soft tissue adaptation has not been addressed in this study but play an important role. Implantoplasty is above all performed to counter microbial challenges, and its impact on preventing bacterial recolonization and facilitating removal of bacterial colonization is considered pivotal to healing and homeostasis of peri-implant health following implantoplasty treatment in response to peri-implantitis challenges. So far in vitro studies on implantoplasty have focused on surface roughness [8–10], heat generation [11–13], and fracture resistance [14, 16, 17]. This study provides some new insights on the soft tissue component following experimental implantoplasty. The establishment of a healthy soft tissue adaptation to the implant surface may be an important part of implantoplasty. Obtaining the smoothest surface possible may hence not be the ultimate goal of implantoplasty, if the soft tissue adaptation can be improved without deterioration of bacterial considerations. A number of studies on implantoplasty have been published in previous years, but it remains as a controversial therapy. There is limited scientific evidence to support an effect on the course of peri-implant diseases. Furthermore, the procedure leads to the release of titanium debris in vast quantities to the peri-implant tissues, which may have adverse biological effects [38]. For this reason, only the supracrestal parts of the implant exposed following bone loss due to peri-implantitis, or as a result of mucosal recessions should be carefully considered for implantoplasty therapy.

This study has noteworthy limitations. Collecting the coins following culture required turning the 96-well plates upside down. Consequently, few coin cell layers were partly damaged. Only intact areas of the coins were used for confocal pictures and analysis. Other limitations include the use of titanium coins, which clearly differ from the cylindrical implants used in patients, and lack of standardization of parameters such as pressure and alignment during the experimental implantoplasty procedure. Although efforts were made to collect the experimental implantoplasty debris, particles may have been lost as aerosols during drilling. The fibronectin imaging was not possible to perform with standardized laser strength in all cases, which may have influenced the subsequent arbitrary quantification. Three of the cytokines were below the detection limit in the immunoassay analysis. No further attempts were made to adapt the cell medium to reach the detection limit. Furthermore, RT-PCR would have been useful in this study in order to verify the cytokine findings in this study also at mRNA level. Attempts were made to measure the total surface area after the experimental implantoplasty but this required the use of a mathematical model and assumptions we could not make. The different cellular behavior observed in this study may indeed also be explained by surface texture parameters not assessed in this study or non-topographical factors such as the altered chemistry of the surface following experimental implantoplasty as demonstrated in the present study. Another factor which may have influenced the results is corrosion from the titanium coins. This study was not designed to identify corrosion and therefore we cannot rule out any titanium corrosion from the coins and potential impact on cells during the 30 days experiment. This needs to be addressed in future research.

## Conclusions

In conclusion, all CB sequences rendered smoother surfaces than DB sequences, and the additional use of GB resulted in smoother surfaces than Ark when combined with both CB and the DB sequences. The different bur sequences did affect the elemental composition of the titanium surface. This study shows that the surface roughness following implantoplasty plays a role in the initial growth of fibroblasts, with the surface roughness S_a_ value showing a weak to moderate negative correlation to the HGF growth. The null hypothesis was rejected. Beyond the first week, fibroblasts flourished on all implantoplasty-treated coins. As compared to the SLA surface every implantoplasty procedure assessed in this study in general led to lower levels of the cytokines VEGF, IL-6, MCP1, MCP3 and IP-10 secreted per fibroblast to the cell medium, and lower levels of fibronectin at 30 days.

## Data Availability

The datasets used and/or analysed during the current study are available from the corresponding author on reasonable request.

## Abbreviations

CB + Ark: Carbide burs + Arkansas stone
CB + BG: Carbide burs + Brownie and Greenie silicone burs
CB: Carbide cutting burs
DB + Ark: Diamond burs and Arkansas stone
DB + BG: Diamond burs + Brownie and Greenie silicone burs
DB: Diamond burs
EDX: Energy dispersive x-ray spectroscopy
HGF: Human Gingival Fibroblasts
POL: Polished coins
S_a_: Arithmetical-mean-height of the surface
SEM: Scanning Electron Microscope
SLA: Coins with SLA surface
S_pk_: Core roughness depth
S_q_: Root-mean-square deviation of the surface
S_z_: Ten-point-height of the surface
